# Prevention of Childhood Adversities and Children’s Common Mental Disorders and School Grades

**DOI:** 10.1001/jamanetworkopen.2023.36408

**Published:** 2023-10-05

**Authors:** Matthias Pierce, Yushi Bai, Alicia Nevriana, Christina Dalman, Holly F. Hope, Kyriaki Kosidou, Anna Ohlis, Susanne Wicks, Kathryn M. Abel

**Affiliations:** 1 Centre for Women’s Mental Health, Division of Psychology and Mental Health, Faculty of Biology, Medicine and Health, University of Manchester, Manchester, United Kingdom; 2 Department of Global Public Health, Karolinska Institutet, Stockholm, Sweden; 3 Unit of Occupational Medicine, Institute of Environmental Medicine, Karolinska Institutet, Stockholm, Sweden; 4 The Center for Epidemiology and Community Medicine, Stockholm, Sweden; 5 Greater Manchester Mental Health NHS Foundation Trust, Manchester, United Kingdom

## Abstract

**Importance:**

Adversity during childhood can limit children’s chances of achieving their optimal developmental and psychological outcomes. Well-designed observational studies might help identify adversities that are most implicated in this, thereby helping to identify potential targets for developing interventions.

**Objective:**

To compare the association between preventing childhood poverty, parental mental illness and parental separation, and the population rate of offspring common mental disorders (ages 16-21 years) or average school grades (age 16 years).

**Design, Setting, and Participants:**

A population-based, longitudinal cohort study using Swedish registries was conducted. A total of 163 529 children born in Sweden between January 1, 1996, and December 31, 1997, were followed up until their 21st birthday. They were linked to registries using Sweden’s national personal identification number. Children were linked to birth parents, hospital records, and school data. Parents were linked to registries containing health, income, sociodemographic, and obstetric data. Analyses were conducted between January 10, 2021, and August 26, 2022.

**Exposures:**

Childhood adversities of relative poverty (household disposable income <50% of the median), parental inpatient admission for a mental illness, or parental separation. Adversities were categorized into developmental periods: ages 0 to 3, 4 to 7, 8 to 11, and 12 to 16 years.

**Main Outcomes and Measures:**

The main outcomes were children’s hospital records with a diagnosis of anxiety or depression between ages 16 and 21 years and school grades at the end of compulsory education (age 16 years). The parametric g-formula modeled population changes in outcomes associated with the counterfactual, hypothetical preventing adversity exposures, accounting for fixed and time-varying confounders. Adjustments were made for parental demographic characteristics, obstetric variables, and socioeconomic data at birth.

**Results:**

A total of 163 529 children were included in the cohort (51.2% boys, 51.4% born in 1996). Preventing all adversities was associated with an estimated change in the prevalence of offspring common mental disorders from 10.2% to 7.6% and an improvement in school grades with an SD of 0.149 (95% CI, 0.147-0.149). Preventing parental separation provided for the greatest improvement, with an estimated 2.34% (95% CI, 2.23%-2.42%) fewer children with a common mental disorder and an improvement in school grades by 0.127 SDs (0.125-0.129). Greater improvements were shown by hypothetically targeting adolescents (age 12-16 years) and those whose parents had a mental illness when the child was born.

**Conclusions and Relevance:**

The results of this cohort modeling study suggest that preventing childhood adversity could provide notable improvements in the rates of common mental disorders and school grades. Many children might achieve better life outcomes if resources are properly allocated to the right adversities (parental separation), the right groups (children with parental mental illness), and at the right time (adolescence).

## Key Points

**Question:**

Is prevention of childhood adversity associated with the prevalence of anxiety or depression and children's school grades?

**Findings:**

In this cohort study identifying 163 529 children using Swedish registries, modeling the prevention of adversities resulted in an estimated 2.6% fewer children in the population with anxiety or depression by age 21 years. In addition, an improvement in school grades at age 16 years was noted.

**Meaning:**

The findings of this modeling study suggest that strategies that target the right adversities (parental separation), at the right time (adolescence), and the right groups (children with parental mental illness) might improve children’s outcomes in the population.

## Teaser

This cohort modeling study estimates changes in the prevalence of anxiety or depression and change in school grades associated with prevention of childhood adversities.

## Introduction

Children who grow up in families with adversity tend to have markedly worse health and social outcomes than children who do not.^1,2,3^ Some adversities are relatively common and easy to identify, for example, having a parent with a mental illness^4^ or substance use disorder,^5^ living in poverty and experiencing material deprivation,^6^ and residential instability or parental separation.^7^ These and other, rarer, and less easily identified adversities (eg, neglect or physical and sexual abuse)^8^ are related to poorer outcomes across a number of domains, including, but not limited to, poorer mental^4^ and physical health,^2^ premature mortality,^9^ lack of school readiness, and poorer educational attainment.^10^

Childhood adversities have been the focus of public health and policy interventions, whether targeted at the adversity itself (eg, welfare payments to alleviate childhood poverty^11^) or intervening on downstream effects (eg, counseling offered to children whose parents separated^12^). When resources are limited, there is a need to understand which adversities are likely to have the most harmful effects on children. Randomized trials are most suited to quantifying causal effects and have been used to establish the effect of some childhood adversities, such as childhood institutionalization^13^ or food insecurity.^14^ However, randomized trials for childhood adversities are rarely ethical and often unfeasible.

In the absence of evidence from randomized trials, high-quality longitudinal, observational studies may help identify exposures that ought to be targeted.^15,16^ In this cohort study, using Sweden’s population registries, we compared the public health outcomes preventing specific childhood adversities: family poverty, parental mental illness, and parental separation. We did this by comparing children’s common mental disorders or school grades under 2 scenarios: one in which, hypothetically, we intervene to prevent childhood adversities, and the other in which we take no action. Therefore, our effect measure can be interpreted as what would happen if we were to hypothetically prevent all children from experiencing that adversity. This measure is directly relevant to public health; therefore, preventing common adversities with a small individual effect size estimate may have a greater impact than preventing low-prevalence adversities with a large individual effect size estimate. We estimated this using the parametric g-formula, which can account for time-dependent confounders that are on the pathway between exposure and outcome.^17^ First, we examined what might happen to children’s outcomes if we were to prevent childhood adversities (occurring at any age) in the Swedish population. Second, we examined whether there are age periods during which preventing adversities may result in the greatest benefit. Third, we examined whether girls or boys might benefit most or whether preventing adversities may be more beneficial in a higher-risk subset of children who, at birth, are exposed to serious parental mental illness.

## Methods

### Data Sources

For this cohort study, 186 032 children born in Sweden between 1996 and 1997 were identified in the Total Population Registry. This registry includes information on all Swedish residents and provides a unique identifier and linkage to their birth mother and father. Parents and children were linked to the immigration and emigration registers. They were also linked to the National Patient Register that records all inpatient psychiatric episodes from 1973 onward. In addition, children were linked to outpatient psychiatric episodes from 2005 onward. Parents were linked to the longitudinal integrated database for health insurance and labor market studies that records annual socioeconomic data, including household income.^18^ In addition, children were linked to the National Medical Birth Register and to the school register that records average grade scores at the end of compulsory education (age 16 years). This study followed the Reporting of Studies Conducted Using Observational Routinely-Collected Health Data (RECORD) reporting guidelines, which is an extension of the Strengthening the Reporting of Observational Studies in Epidemiology (STROBE) reporting guideline. The study was approved by the regional ethical review board of the Karolinska Institutet, Stockholm, Sweden.

**Figure 1.**
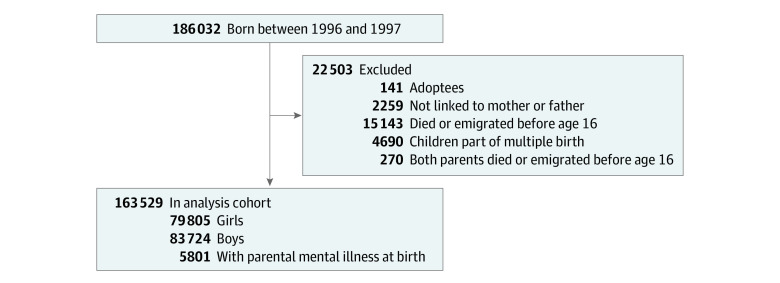
Selection Into Cohort

The following were excluded from the analysis (in turn): adoptees (n = 141), children who could not be linked to either birth mother or father (n = 2259), children who died or emigrated before the age of 16 years ( = 15 143), children who were part of a multiple birth (n = 4960), or children whose parents died or emigrated before the age of 16 years (n = 270). This resulted in a final analysis sample of 163 529 children (Figure 1).

### Indicators of Childhood Adversity

Three childhood adversities were defined during ages 0 to 16 years: having a mother or father with a serious mental disorder, as indicated by an inpatient hospitalization; living in a household in relative poverty (using the Organisation for Economic Co-operation and Development definition of households whose disposable income is below half the median household disposable income in the total population^19^); or having parents who were separated, defined as parents being assigned different household identifiers. Adversities occurring over childhood were categorized into key developmental periods: 0 to 3 years representing infancy and early development, 4 to 7 years representing preschool; 8 to 11 years representing preadolescence; and 12 to 16 years representing adolescence.

### Outcomes

Two outcomes were considered. The first was the children’s final school grades at age 16 years following the end of compulsory education; these were centered and divided by the sample SD to convert them to *z* scores. The second was whether the child had an inpatient or outpatient hospital episode, up to December 31, 2015, for a common mental disorder (anxiety or depression) occurring between the ages of 16 and 21 years, as indicated by an *International Statistical Classification of Diseases and Related Health Problems, Tenth Revision* code indicating depression (F32-34, F38-39, excluding F32.3 and F33.3, with associated psychosis) or anxiety- and stress-related disorders (F40-45, F48).

### Covariates

The aim of the analysis was to quantify whether preventing adversities over childhood is associated with outcomes for children. The study aim relates to changes across childhood, irrespective of factors that might have been present at birth. Therefore, baseline variables were included in the analysis if they were either likely to be associated with both exposure and outcome (eg, parental demographic characteristics) or on the pathway between putative unobserved confounders and the outcome (eg, obstetric variables). In addition, time-dependent variables were included if they changed over follow-up and were considered associated with future instances of an adversity and the outcome. Further reasoning behind the selection of control variables is provided in eMethods 1 in Supplement 1. Thus, the following time-invariant baseline variables were selected from those available in the Swedish registers: highest level of education of the mother or father (secondary, postsecondary, and degree), serious parental mental illness (any inpatient hospital record with a mental disorder in the 5 years before birth), parental age, parental country of birth (Sweden, other Scandinavian, African, Asian, other European, Middle Eastern, and other), household income at birth, birth weight, gestational age, and year of birth of the child. In addition, the number of siblings was included as a time-dependent variable and all childhood adversity variables were considered as time-dependent confounders for each other.

### Statistical Analysis

The estimand of interest was the population intervention effect size^20^; that is, the difference between the expected outcome if an adversity were prevented with the expected outcome when no action is taken (the natural course). This was estimated using the parametric g-formula, which is a generalization of standardization to longitudinal data that simulates what would happen under what-if counterfactual scenarios.^17^ It was implemented by fitting regression models to each time-dependent confounder and to the outcome variables. Using these models, data were simulated in time sequence, based on hypothetical scenarios (eg, setting parental separation to 0 for all children in the sample). For resulting estimates to be identified, the assumptions of conditional exchangeability, consistency, and positivity must be met (eMethods 2 in Supplement 1). The method was implemented using the gfoRmula package^21^ in R, version 4.0.2 (R Foundation for Statistical Computing).

Within the g-formula algorithm, binary variables were modeled using logistic regression and continuous variables were modeled using linear regression. All models included baseline variables for parental demographic characteristics, parental mental illness in the 5 years before birth, income at birth, and obstetric variables (eMethods 1 in Supplement 1 provides details of variables chosen and rationale). In addition, models included the time-updated variable for number of siblings born during childhood. All models beyond the first time point included a lagged version of all time-updated variables (ie, adversities and number of siblings). In addition, they included a variable for the average mean household disposable income for each period. Continuous covariates (income, birth weight, and parental age) were converted into cubic splines to account for nonlinear relationships.

Several hypothetical scenarios were considered, corresponding to maximal intervention strategies that involve preventing childhood adversities. The first was to prevent maternal mental illness (as measured by an inpatient admission) during all age periods and the second was to prevent paternal mental illness during all age periods. The third was to prevent any household from going into relative poverty (defined as falling below 50% of the median household income). The fourth was to prevent parental separation. The outcome of preventing all of these adversities was also determined, as well as preventing all adversities at specific age periods (0-3, 4-7, 8-11, and 12-16 years). More information on the hypothetical prevention scenarios is available in eMethods 3 in Supplement 1. The effect size of these was estimated by subtracting the outcome under each scenario with the expected value of the outcome when we do not intervene (termed the natural course). Confidence intervals were calculated using 500 bootstrap samples. Analyses were conducted between January 10, 2021, and August 26, 2022. Our interpretation of the results was based on the effect size and width of the 95% CIs.

## Results

A total of 163 529 children born in Sweden between January 1, 1996, and December 31, 1997, were included in the cohort (48.8% girls, 51.2% boys, 3.6% with a parent with mental illness at birth, 51.4% born in 1996). The majority (109 021 [66.7%]) experienced at least 1 adversity over childhood. Children who experienced adversity tended to be more likely than others to have parents with lower levels of education, to have younger (<25 years) or older (≥40 years) parents, to have parents born outside of Sweden, and to have older siblings (Table 1). Children with adversity over childhood were marginally more likely to be born preterm or with low birth weight.

**Table 1.** Demographic Characteristics

Characteristic	Any adversity over childhood, No. (%)	
	Yes (n = 104 989)	No (n = 58 540)
Year of birth		
1996	54 066 (51.5)	29 934 (51.1)
1997	50 923 (48.5)	28 606 (48.9)
Age of mother at birth, y		
<21	5548 (5.3)	311 (0.5)
19-24	19 519 (18.6)	5774 (9.9)
25-29	36 634 (34.9)	23 362 (39.9)
30-39	41 008 (39.1)	27 822 (47.5)
≥40	2280 (2.2)	1271 (2.2)
Age of father at birth		
<21 y	1757 (1.7)	34 (0.1)
21-24	10 204 (9.7)	2467 (4.2)
25-29	30 170 (28.7)	17 456 (29.8)
30-39	51 726 (49.3)	33 583 (57.4)
≥40	11 131 (10.6)	5000 (08.5)
Maternal education		
Pre–upper secondary	12 680 (12.1)	1615 (02.8)
Secondary	54 768 (52.2)	25 981 (44.4)
Post–upper secondary	37 313 (35.5)	30 940 (52.9)
Missing	228 (0.2)	4
Paternal education		
Pre–upper secondary	17 472 (16.6)	4571 (07.8)
Secondary	59 701 (56.9)	28 952 (49.5)
Post–upper secondary	27 555 (26.3)	24 986 (42.7)
Missing	261 (0.3)	31 (0.1)
Country of birth, mother		
African	1982 (1.9)	84 (0.1)
Asian	3030 (2.9)	492 (0.8)
European, other	5784 (5.5)	1020 (1.7)
Middle Eastern	6609 (6.3)	316 (0.5)
Other	1512 (1.4)	324 (0.6)
Scandinavian, other	2658 (2.5)	1232 (2.1)
Sweden	83 414 (79.5)	55 072 (94.1)
Country of birth, father		
African	2210 (2.1)	83 (0.1)
Asian	2355 (2.2)	212 (0.4)
European, other	6326 (6.0)	1054 (1.8)
Middle Eastern	7786 (7.4)	422 (0.7)
Other	1806 (1.7)	272 (0.5)
Scandinavian, other	2621 (2.5)	1067 (1.8)
Sweden	81 884 (78.0)	55 430 (94.7)
Missing	1	0
Birth order		
1st	44 242 (42.1)	26 735 (45.7)
2nd	37 474 (35.7)	24 450 (41.8)
3rd or higher	23 273 (22.2)	7355 (12.6)
Prior parental mental illness^a^		
None	100 064 (95.3)	57 664 (98.5)
Paternal only	1883 (1.8)	337 (0.6)
Maternal only	2829 (2.7)	535 (00.9)
Both parents	213 (0.2)	4
Relative poverty at birth	17 753 (16.9)	293 (0.5)
Born low birth weight^b^	3159 (3.0)	1600 (2.7)
Born preterm^c^	5071 (4.8)	2579 (4.4)

^a^ Defined as any inpatient episode with a mental illness diagnosis in the 5 years prior to birth.

^b^ Defined as less than 2500 g.

^c^ Defined as gestational age of less than 37 weeks.

**Figure 2.**
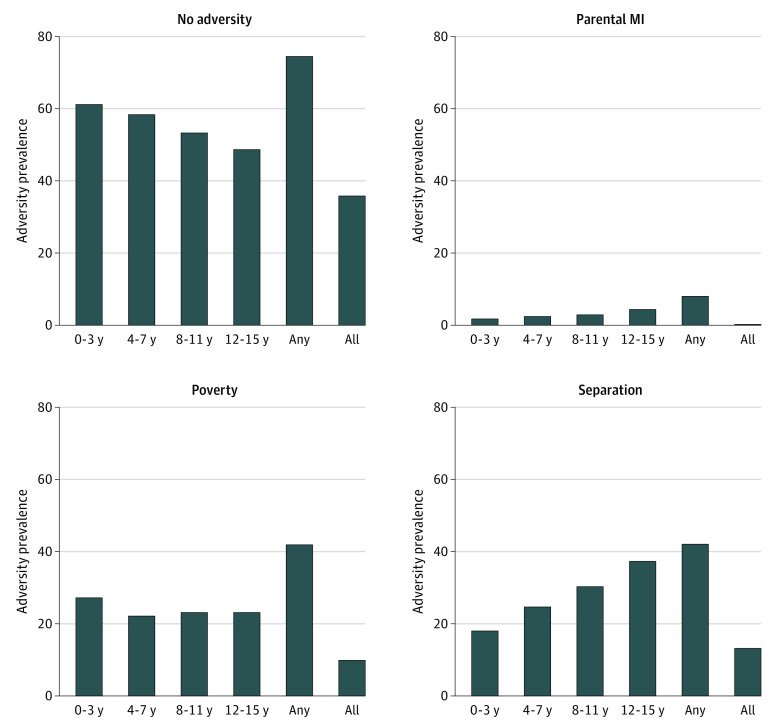
Prevalence of Adversity by Developmental Period MI indicates mental illness.

Over childhood, relative poverty and parental separation were considerably more common than having a parent with an inpatient admission with a serious mental illness (Figure 2). The prevalence of serious parental mental illness and parental separation increased over childhood, while the rate of poverty was highest for children of younger ages. Those whose parent had been admitted with a mental illness over childhood had considerably higher rates of a common mental disorder from age 16 to 21 years (eFigure 1 in Supplement 1) and lower school grades at age 16 years (eFigure 2 in Supplement 1) than children exposed to other adversities or not exposed to any adversity. Children exposed to an adversity across all age groups tended to have worse outcomes than children exposed during a single age group.

An estimated 2.6% of children would be prevented from having a common mental disorder if all adversities were prevented (95% CI, 2.5%-2.7%) (Table 2) and their school grades would improve by 0.149 of an SD (95% CI, 0.147-0.149). All scenarios in which adversities were prevented resulted in an estimated reduction in the population prevalence of common mental disorders between the ages of 16 and 21 years (from 10.2% to 7.6%) and an improvement in school grades at age 16 years with an SD of 0.149 (95% CI, 0.147-0.149). However, some of the effect sizes were marginal (Table 2). The greatest improvement, except preventing all adversities, came from preventing parental separation, which was associated with 2.34% (95% CI, 2.23%-2.42%) fewer children experiencing a common mental disorder and an improvement in average school grades by 0.127 SDs (95% CI, 0.125-0.129). Preventing parental mental illness hospitalization resulted in a greater improvement in the prevalence of common mental disorders than preventing household poverty; however, preventing poverty resulted in a marginally greater improvement in school grades. Preventing adversities in adolescence was estimated to result in greater improvement to either the population rate in common mental disorders or school grades than intervening in any other age period.

**Table 2.** Results From Parametric g-Formula Showing Prevalence of Common Mental Illness and Mean School Grades

Scenario	Common mental disorder		School grades at age 16 y	
	Prevalence, %	Reduction, % (95% CI)	Mean (*z* score)	Improvement, SD (95% CI)
Natural course	10.2	1 [Reference]	0.000	1 [Reference]
Prevent parental mental illness	9.7	0.41 (0.40-0.41)	0.009	0.009 (0.006-0.008)
Prevent household relative poverty	10.1	0.10 (0.08-0.10)	0.018	0.017 (0.017-0.018)
Prevent parental separation	7.8	2.34 (2.23-2.42)	0.127	0.127 (0.125-0.129)
Prevent all adversities	7.6	2.60 (2.47-2.68)	0.150	0.149 (0.147-0.149)
Prevent all adversities at specific ages				
0-3 y	9.6	0.58 (0.52-0.62)	0.034	0.033 (0.031-0.034)
4-7 y	8.9	1.27 (1.16-1.37)	0.072	0.071 (0.067-0.074)
8-11 y	8.4	1.73 (1.56-1.87)	0.090	0.089 (0.083-0.094)
12-16 y	7.4	2.70 (2.53-2.86)	0.118	0.117 (0.110-0.123)

Considering models applied to subgroups, preventing adversities in higher-risk children with preexisting parental mental illness was associated with the largest reduction in the rate of common mental disorders and the greatest improvement in school grades (Table 3). For example, the estimated rate of common mental disorders for this group was reduced from 19.3% (95% CI, 18.4%-20.2%) to 13.4% (95% CI, 11.9%-14.9%) if we were to intervene in all adversities—a reduction of 5.9% (95% CI, 4.6%-7.2%). There was also evidence that preventing adversities had a higher effect estimate size in the rate of common mental disorders in girls than boys; in particular, intervening in maternal mental illness or parental separation had a higher effect size estimate for the risk of common mental disorders in girls than boys.

**Table 3.** Effect Size Estimates From Subgroup Analysis

Subgroup and intervention	Value (95% CI)			
	Common mental disorder		School grades	
	Prevalence, %	Reduction, %	Mean (*z* score)	Improvement
**Girls**				
Natural course	13.2 (12.9 to 13.4)	1 [Reference]	0.196 (0.188 to 0.202)	1 [Reference]
Prevent parental mental illness hospitalization	12.7 (12.4 to 12.9)	0.49 (0.42 to 0.56)	0.205 (0.197 to 0.212)	0.009 (0.007 to 0.011)
Prevent household poverty	13.0 (12.8 to 13.3)	0.13 (0.09 to 0.17)	0.212 (0.205 to 0.218)	0.016 (0.015 to 0.017)
Prevent parental separation	10.3 (10.0 to 10.5)	2.90 (2.68 to 3.12)	0.322 (0.314 to 0.330)	0.126 (0.122 to 0.132)
Prevent all adversities	9.9 (9.6 to 10.2)	3.24 (3.02 to 3.47)	0.344 (0.336 to 0.352)	0.148 (0.142 to 0.154)
**Boys**				
Natural course	7.3 (7.1 to 7.5)	1 [Reference]	−0.213 (−0.219 to −0.205)	1 [Reference]
Prevent parental mental illness hospitalization	7.0 (6.7 to 7.1)	0.35 (0.28 to 0.41)	−0.204 (−0.211 to −0.196)	0.008 (0.006 to 0.010)
Prevent household poverty	7.2 (7.0 to 7.4)	0.08 (0.05 to 0.12)	−0.195 (−0.201 to −0.187)	0.018 (0.016 to 0.020)
Prevent parental separation	5.5 (5.3 to 5.7)	1.81 (1.63 to 1.95)	−0.087 (−0.094 to −0.078)	0.126 (0.120 to 0.131)
Prevent all	5.3 (5.1 to 5.5)	2.01 (1.84 to 2.16)	−0.064 (−0.072 to −0.055)	0.149 (0.142 to 0.154)
**Children with parental mental illness**				
Natural course	19.3 (18.4 to 20.2)	1 [Reference]	−0.427 (−0.456 to −0.398)	1 [Reference]
Prevent parental mental illness hospitalization	17.5 (16.5 to 18.5)	1.78 (1.21 to 2.41)	−0.411 (−0.447 to −0.381)	0.016 (−0.004 to 0.031)
Prevent household poverty	19.2 (18.2 to 20.1)	0.12 (−0.08 to 0.36)	−0.399 (−0.429 to −0.371)	0.027 (0.022 to 0.033)
Prevent parental separation	14.5 (13.0 to 16.1)	4.85 (3.49 to 6.05)	−0.216 (−0.258 to −0.179)	0.211 (0.174 to 0.243)
Prevent all	13.4 (11.9 to 14.9)	5.94 (4.60 to 7.16)	−0.183 (−0.230 to −0.145)	0.243 (0.202 to 0.278)

## Discussion

As reported elsewhere, children raised in an environment with parental mental illness, household poverty, and parental separation have considerably poorer school grades and higher rates of anxiety or depression than children who do not experience these adversities.^2,3,22^ We estimate that preventing these adversities was associated with 2.6% fewer children in the population developing a common mental disorder between the ages of 16 and 21 years, accounting for a third of cases, and an estimated 3000 children per year in Sweden (at the current birth rate). We also estimate that preventing these adversities was associated with an improvement in school grades by 0.15 of an SD. For comparison, the adjusted difference between children whose father had further education beyond compulsory schooling, compared with those whose education ended at the compulsory level was 0.19 of an SD (eTable in Supplement 1).

Of all measured adversities, preventing the outcomes noted with parental separation was associated with the greatest reduction in the prevalence of common mental disorders and the greatest improvement in school grades at age 16 years. This was despite the fact that children with a parent hospitalized with a mental illness had poorer outcomes. This is because, in Sweden, parental separation was a far more common adversity, affecting 42% of children at some point over childhood, compared with only 8% of children having a parent with a serious enough mental illness to require hospitalization. Other studies have also shown that parental separation negatively affects children’s mental health or educational attainment.^23,24^ However, to our knowledge, this is the first study to use population measures to compare parental separation and other adversities.

We noted only marginal changes associated with preventing households developing relative poverty. This is counter to many reports showing childhood poverty is associated with poor outcomes.^25^ Unlike most studies, however, our analysis used models that can consistently adjust for time-varying confounders. Our analysis more closely agrees with a recent study using Finnish registries that, using a sibling-discordance design, provides little evidence of an association between poverty and children’s mental health outcomes.^26^ It is notable that this study also comes from a Scandinavian setting where larger welfare systems and lower levels of income inequality compared with most countries may provide resilience against the effects of relative poverty.^27^

We provide evidence that preventing adversities during adolescence might be more beneficial than preventing adversities during younger ages. This may be counter to expectations about childhood being the most sensitive period.^28,29^ However, we note that the likelihood of adversities, such as parental separation, increased during childhood and were most common during adolescence. Therefore, our findings reflect that preventing all adversities during this period may be associated with the prevalence of anxiety or depression and school grades, and our results are consistent with another study that took a public health perspective.^30^

This investigation provides evidence that children at high risk may derive the greatest benefit if adversities are prevented. According to the model, children with serious parental mental illness may benefit more from preventing adversities than children in the whole sample. For example, an estimated 5.9% (95% CI, 4.6%-7.2%) of children with parental mental illness would be prevented from developing a common mental disorder if they were no longer exposed to all these adversities, compared with 2.6% of the whole sample (95% CI, 2.5%-2.7%). However, because only 3.6% of children had evidence of serious parental mental illness (inpatient admission in 5 years prior to child’s birth), this approach has a smaller benefit in the population overall. This is only one perspective when allocating resources. Assessment of whether high-risk approaches are desirable needs to include many perspectives, such as the existence of evidence-based methods in the community setting that would prevent the outcomes of an adversity as well as the relative number of children who would benefit and the additional costs of targeting and intervening with high-risk compared with all children.^31^

### Limitations

This study has several limitations. First, the registers lack data on many confounding variables that might contribute to risk of an adversity occurring and children’s developmental outcomes. These include measures of domestic violence,^32^ social support outside parents (eg, from grandparents^33^), or genes that influence mental illness risk and severity.^34^ However, the adversities chosen are all broad indicators of both social and genetic load, for example, parental mental disorders affect the child both socially and genetically. Second, some adversities are quite crudely measured. Parental mental illness was only measured using Sweden’s inpatient registry, as their outpatient and primary care registries were not available for the whole follow-up period. Inpatient admissions for mental illness only accounts for 20% of treated mental illnesses in Sweden,^35^ albeit the most severe. Poverty was measured using income relative to the median value. However, this ignores other dimensions of poverty, including material wealth, housing, and job insecurity. Third, the outcome of common mental disorders, while using both inpatient and outpatient registries, still only captures a proportion of cases and the likelihood that a younger person seeks care for mental health problems is dependent on a range of factors, including gender and social class. Fourth, the measures we used are analogous to intervention strategies that would eliminate an adversity, and this will be highly unrealistic in many situations and harmful in others. For example, in families in which there is violence, parental separation may be the best outcome. Therefore, our estimates should be taken as indicative of potential targets for intervention, and future analyses should incorporate information about the extent that these realistically change adversities.

## Conclusions

Several implications for public health and policy are raised in this cohort modeling study. First, based on these models, if resources are properly allocated at the right adversities, at the right time, and at the right group, then many children could be prevented from becoming mentally ill, and we could boost their chances of achieving good school grades. Second, our findings suggest that resources should be diverted toward supporting families at an early stage of parental separation, for example, family-based support at child health care centers. Prior research has suggested that interparental conflicts explain a substantial part of the negative effect^36^ associated with parental separation; thus, interventions that improve children’s coping with transitioning through parental separation may be most beneficial.^12^ Third, our finding that adolescence appears to be a critical period for intervention needs further research. We know that the transition to adulthood is a sensitive time both for parents and children; however, many interventions have been targeted at early years. Getting this right is critical, especially in light of the very large numbers of adolescents and young people now presenting to services with mental illness. Fourth, when resources are limited, intervening in families with serious parental mental illness may be a useful strategy.

## Notes

Supplement 1. **eMethods 1.** Further Details on Causal Model **eMethods 2.** Further Details on Underlying Assumptions Behind the Causal Model **eMethods 3.** Hypothetical Prevention Scenarios **eTable.** Association Between Baseline Covariates and CMD Prevalence or School Leaving Grades **eFigure 1.** Prevalence of Common Mental Disorders by Adversity Exposure **eFigure 2.** Average School Grade *z* Scores by Adversity Exposure
eReferences

Supplement 2.
Data Sharing Statement

## References

[1] Adjei NK, Schlüter DK, Straatmann VS, ; ORACLE consortium. Impact of poverty and family adversity on adolescent health: a multi-trajectory analysis using the UK Millennium Cohort Study. Lancet Reg Health Eur. 2021;13:100279. doi:10.1016/j.lanepe.2021.100279 35199082 PMC8841277

[2] Hughes K, Bellis MA, Hardcastle KA, . The effect of multiple adverse childhood experiences on health: a systematic review and meta-analysis. Lancet Public Health. 2017;2(8):e356-e366. doi:10.1016/S2468-2667(17)30118-4 29253477

[3] Grummitt LR, Kreski NT, Kim SG, Platt J, Keyes KM, McLaughlin KA. Association of childhood adversity with morbidity and mortality in US adults: a systematic review. JAMA Pediatr. 2021;175(12):1269-1278. doi:10.1001/jamapediatrics.2021.2320 34605870 PMC9059254

[4] Lewis G, Neary M, Polek E, Flouri E, Lewis G. The association between paternal and adolescent depressive symptoms: evidence from two population-based cohorts. Lancet Psychiatry. 2017;4(12):920-926. doi:10.1016/S2215-0366(17)30408-X 29153626

[5] Berg L, Bäck K, Vinnerljung B, Hjern A. Parental alcohol-related disorders and school performance in 16-year-olds—a Swedish national cohort study. Addiction. 2016;111(10):1795-1803. doi:10.1111/add.13454 27178010 PMC5089658

[6] Subramanyam MA, Kawachi I, Subramanian SV. Reactions to fair society, healthy lives (the Marmot Review). Soc Sci Med. 2010;71(7):1221-1222. doi:10.1016/j.socscimed.2010.07.002 20685020

[7] Björkenstam E, Dalman C, Vinnerljung B, Weitoft GRR, Walder DJ, Burström B. Childhood household dysfunction, school performance and psychiatric care utilisation in young adults: a register study of 96 399 individuals in Stockholm County. J Epidemiol Community Health. 2016;70(5):473-480. doi:10.1136/jech-2015-206329 26646690

[8] LeMoult J, Humphreys KL, Tracy A, Hoffmeister JA, Ip E, Gotlib IH. Meta-analysis: exposure to early life stress and risk for depression in childhood and adolescence. J Am Acad Child Adolesc Psychiatry. 2020;59(7):842-855. doi:10.1016/j.jaac.2019.10.011 31676392 PMC11826385

[9] Claussen B, Davey Smith G, Thelle D. Impact of childhood and adulthood socioeconomic position on cause specific mortality: the Oslo Mortality Study. J Epidemiol Community Health. 2003;57(1):40-45. doi:10.1136/jech.57.1.40 12490647 PMC1732268

[10] Shen H, Magnusson C, Rai D, . Associations of parental depression with child school performance at age 16 years in Sweden. JAMA Psychiatry. 2016;73(3):239-246. doi:10.1001/jamapsychiatry.2015.2917 26842307

[11] Bäckman O, Ferrarini T. Combating child poverty? a multilevel assessment of family policy institutions and child poverty in 21 old and new welfare states. J Soc Policy. 2010;39(2):275-296. doi:10.1017/S0047279409990456

[12] Herrero M, Roca P, Cormenzana S, Martínez-Pampliega A. The efficacy of postdivorce intervention programs for children: a meta-analytical review. Fam Process. 2023;62(1):74-93. doi:10.1111/famp.12807 36054156

[13] Humphreys KL, Gleason MM, Drury SS, . Effects of institutional rearing and foster care on psychopathology at age 12 years in Romania: follow-up of an open, randomised controlled trial. Lancet Psychiatry. 2015;2(7):625-634. doi:10.1016/S2215-0366(15)00095-4 26303560 PMC4550037

[14] Burke M, Cabili C, Berman D, Forrestal S, Gleason P. A randomized controlled trial of three school meals and weekend food backpacks on food security in Virginia. J Acad Nutr Diet. 2021;121(1S):S34-S45. doi:10.1016/j.jand.2020.10.026 33342523

[15] Craig P, Dieppe P, Macintyre S, Michie S, Nazareth I, Petticrew M; Medical Research Council Guidance. Developing and evaluating complex interventions: the new Medical Research Council guidance. BMJ. 2008;337(7676):a1655. doi:10.1136/bmj.a1655 18824488 PMC2769032

[16] Black N. Why we need observational studies to evaluate the effectiveness of health care. BMJ. 1996;312(7040):1215-1218. doi:10.1136/bmj.312.7040.1215 8634569 PMC2350940

[17] Robins J. A new approach to causal inference in mortality studies with a sustained exposure period—application to control of the healthy worker survivor effect. Mathematical Modelling. 1986;7(9–12):1393-1512. doi:10.1016/0270-0255(86)90088-6

[18] Ludvigsson JF, Svedberg P, Olén O, Bruze G, Neovius M. The longitudinal integrated database for Health Insurance and Labour Market Studies (LISA) and its use in medical research. Eur J Epidemiol. 2019;34(4):423-437. doi:10.1007/s10654-019-00511-8 30929112 PMC6451717

[19] Society at a Glance 2019: OECD Social Indicators. OECD Publishing. 2019. Accessed September 6, 2023. https://www.oecd-ilibrary.org/social-issues-migration-health/society-at-a-glance-2019_soc_glance-2019-en

[20] Hubbard AE, Laan MJ. Population intervention models in causal inference. Biometrika. 2008;95(1):35-47. doi:10.1093/biomet/asm097 18629347 PMC2464276

[21] McGrath S, Lin V, Zhang Z, . gfoRmula: an R Package for Estimating the Effects of Sustained Treatment Strategies via the Parametric g-formula. Patterns (N Y). 2020;1(3):100008. doi:10.1016/j.patter.2020.100008 32656541 PMC7351102

[22] Robles A, Gjelsvik A, Hirway P, Vivier PM, High P. Adverse childhood experiences and protective factors with school engagement. Pediatrics. 2019;144(2):e20182945. doi:10.1542/peds.2018-2945 31285393

[23] Auersperg F, Vlasak T, Ponocny I, Barth A. Long-term effects of parental divorce on mental health—a meta-analysis. J Psychiatr Res. 2019;119:107-115. doi:10.1016/j.jpsychires.2019.09.011 31622869

[24] Amato PR, Keith B. Parental divorce and the well-being of children: a meta-analysis. Psychol Bull. 1991;110(1):26-46. doi:10.1037/0033-2909.110.1.26 1832495

[25] Ridley M, Rao G, Schilbach F, Patel V. Poverty, depression, and anxiety: causal evidence and mechanisms. Science. 2020;370(6522):eaay0214. doi:10.1126/science.aay0214 33303583

[26] Sariaslan A, Mikkonen J, Aaltonen M, Hiilamo H, Martikainen P, Fazel S. No causal associations between childhood family income and subsequent psychiatric disorders, substance misuse and violent crime arrests: a nationwide Finnish study of >650 000 individuals and their siblings. Int J Epidemiol. 2021;50(5):1628-1638. doi:10.1093/ije/dyab099 34050646 PMC8580272

[27] Jones C, Burström B, Marttila A, Canvin K, Whitehead M. Studying social policy and resilience to adversity in different welfare states: Britain and Sweden. Int J Health Serv. 2006;36(3):425-442. doi:10.2190/E9H5-URYL-2W4U-QED6 16981624

[28] Marini S, Davis KA, Soare TW, . Adversity exposure during sensitive periods predicts accelerated epigenetic aging in children. Psychoneuroendocrinology. 2020;113:104484. doi:10.1016/j.psyneuen.2019.104484 31918390 PMC7832214

[29] Dunn EC, Soare TW, Zhu Y, . Sensitive periods for the effect of childhood adversity on DNA methylation: results from a prospective, longitudinal study. Biol Psychiatry. 2019;85(10):838-849. doi:10.1016/j.biopsych.2018.12.023 30905381 PMC6552666

[30] Manhica H, Straatmann VS, Lundin A, Agardh E, Danielsson AK. Association between poverty exposure during childhood and adolescence, and drug use disorders and drug-related crimes later in life. Addiction. 2021;116(7):1747-1756. doi:10.1111/add.15336 33197093 PMC8247994

[31] Fusar-Poli P, Correll CU, Arango C, Berk M, Patel V, Ioannidis JPA. Preventive psychiatry: a blueprint for improving the mental health of young people. World Psychiatry. 2021;20(2):200-221. doi:10.1002/wps.20869 34002494 PMC8129854

[32] Abel KM, Heuvelman H, Rai D, . Intelligence in offspring born to women exposed to intimate partner violence: a population-based cohort study. Wellcome Open Res. 2019;4:107. doi:10.12688/wellcomeopenres.15270.1 31681855 PMC6820818

[33] Sadruddin AFA, Ponguta LA, Zonderman AL, Wiley KS, Grimshaw A, Panter-Brick C. How do grandparents influence child health and development? a systematic review. Soc Sci Med. 2019;239:112476. doi:10.1016/j.socscimed.2019.112476 31539783

[34] Hannigan LJ, Eilertsen EM, Gjerde LC, . Maternal prenatal depressive symptoms and risk for early-life psychopathology in offspring: genetic analyses in the Norwegian Mother and Child Birth Cohort Study. Lancet Psychiatry. 2018;5(10):808-815. doi:10.1016/S2215-0366(18)30225-6 30245187

[35] Sundquist J, Ohlsson H, Sundquist K, Kendler KS. Common adult psychiatric disorders in Swedish primary care where most mental health patients are treated. BMC Psychiatry. 2017;17(1):235. doi:10.1186/s12888-017-1381-4 28666429 PMC5493066

[36] Lucas N, Nicholson JM, Erbas B. Child mental health after parental separation: the impact of resident/non-resident parenting, parent mental health, conflict and socioeconomics. J Fam Stud. 2013;19(1):53-69. doi:10.5172/jfs.2013.19.1.53

