# Supplementary material for: Prevention of Childhood Adversities and Children’s Common Mental Disorders and School Grades
Source: JAMA Netw Open. 2023 Oct 5;6(10):e2336408. doi: 10.1001/jamanetworkopen.2023.36408 (PMC10556962; doi:10.1001/jamanetworkopen.2023.36408)
Supplement: Supplement 1. — eMethods 1. Further Details on Causal Model eMethods 2. Further Details on Underlying Assumptions Behind the Causal Model eMethods 3. Hypothetical Prevention Scenarios eTable. Association Between Baseline Covariates and CMD Prevalence or School Leaving Grades eFigure 1. Prevalence of Common Mental Disorders by Adversity Exposure eFigure 2. Average School Grade z Scores by Adversity Exposure eReferences [file jamanetwopen-e2336408-s001.pdf]

Supplementary Online Content

Pierce M, Bai Y, Nevriana A, et al. Prevention of childhood adversities and outcomes in mental disorder and school grades. *JAMA Netw Open*. 2023;6(10):e2336408. doi:10.1001/jamanetworkopen.2023.36408

eMethods 1. Further Details on Causal Model

eMethods 2. Further Details on Underlying Assumptions Behind the Causal Model

eMethods 3. Hypothetical Prevention Scenarios

eTable. Association Between Baseline Covariates and CMD Prevalence or School Leaving Grades

eFigure 1. Prevalence of Common Mental Disorders by Adversity Exposure

eFigure 2. Average School Grade *z* Scores by Adversity Exposure

eReferences

This supplementary material has been provided by the authors to give readers additional information about their work.

© 2023 Pierce M et al. *JAMA Network Open.*

eMethods 1: Further details on causal model

The aim of the study was to estimate what would happen to children's outcomes (CMD prevalence or average school leaving grades) when the following childhood adversities were prevented: parent's inpatient admission for mental illness; separation between parents; and household poverty. Each adversity over childhood is confounded by prior instances of that adversity and co-adversities, and any other measured or unmeasured variables that might cause it and the outcome. This is represented in the causal DAG below, for two time periods.

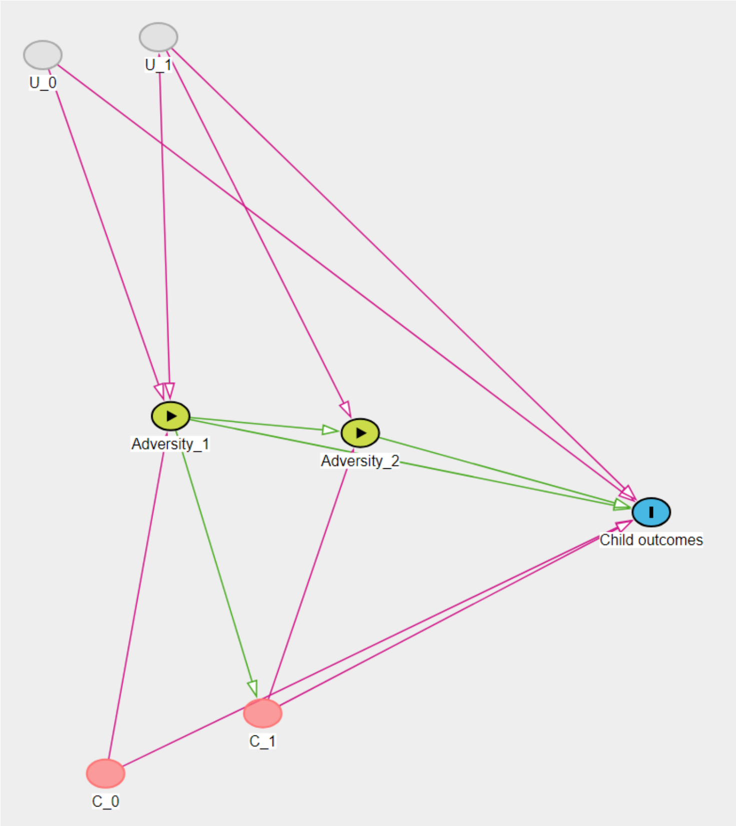

The nodes are:

U_0 = unobserved baseline confounders
C_0 = measured baseline confounders.
Adversity_1 = adversities measured at time 1
U_1 = unmeasured dynamic confounders at time 1.
C_1 = measured dynamic confounders
Adversity_2 = adversities measured at time 2

Example variables for each of these are provided below. This model assumes that part of the effect of adversity at time 1 on children's outcomes is mediated through its effect on a future confounder. For example, part of the effect of parental mental illness on children's outcomes is through its effect on poverty. Controlling for this in a standard regression model is not appropriate because it will remove some of the effect of the exposure, i.e. from T1→C1→Y. G-methods get around this by 'standardising' the analysis, to get the counterfactual distribution of the confounders and outcome, under hypothetical scenarios where the exposure is set to a particular value[1].

In our analysis, adversities were categorised within four developmental age-groups: 0 to 3 years; 4 to 7 years; 8 to 11 years; 12 to 15 years. At each period, all adversity factors were chosen as confounders for each other, as well as adversities measured at the previous time point. The ordering of adversities during a particular period was chosen to reflect likely causal ordering, however it is not necessary that this is correct, the important thing is that the variables are simulated in ways that are seen in the observed data. The following DAG shows the causal model amongst all measured variables.

© 2023 Pierce M et al. *JAMA Network Open.*

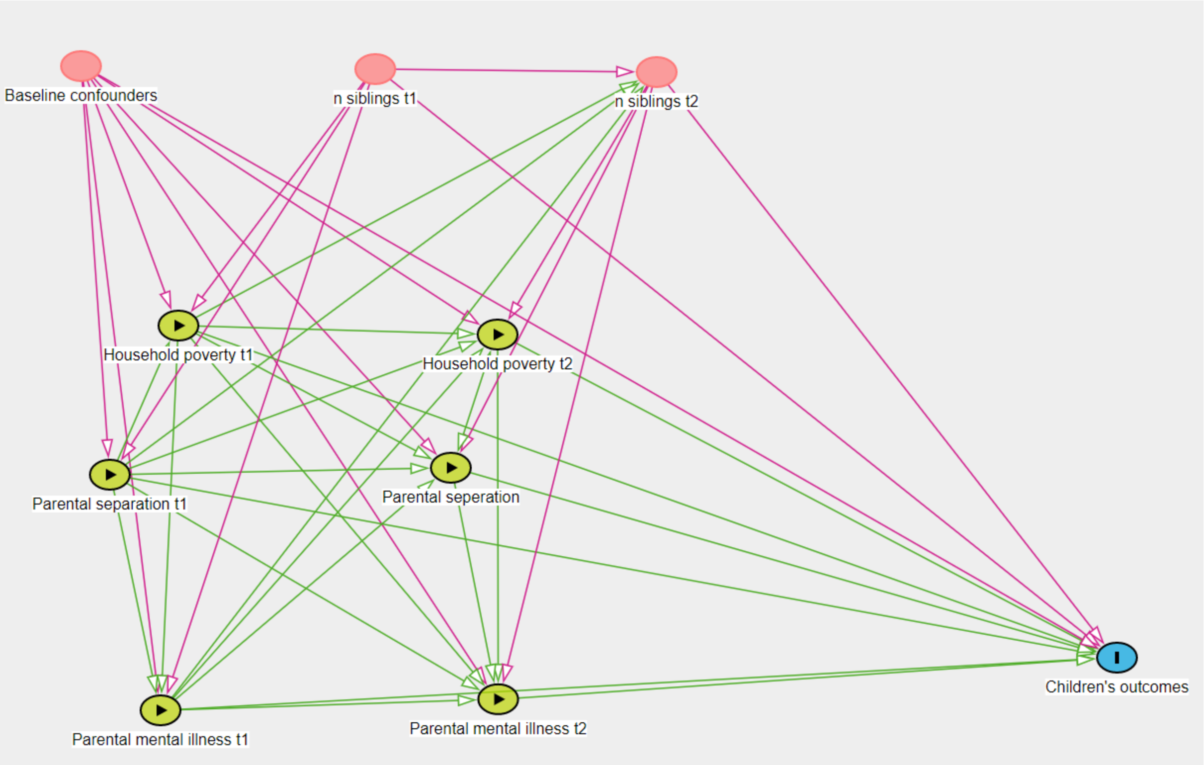

Choice of baseline variables

Variables were conditioned on if they were measured prior to the exposure and were either considered to be confounders, or they were on the pathway between unobserved confounders and the outcome. The DAG below shows specific variables used in this analysis, their relationship between the exposures (adversity) and children's outcomes (mental health risk or school leaving grades). The variables shown in red are those that are measured in the data and have a direct confounding pathway. Variables in grey are unmeasured confounders (U) or co-adversities which cannot be consistently controlled for in the analysis (co-adversities).

© 2023 Pierce M et al. *JAMA Network Open.*

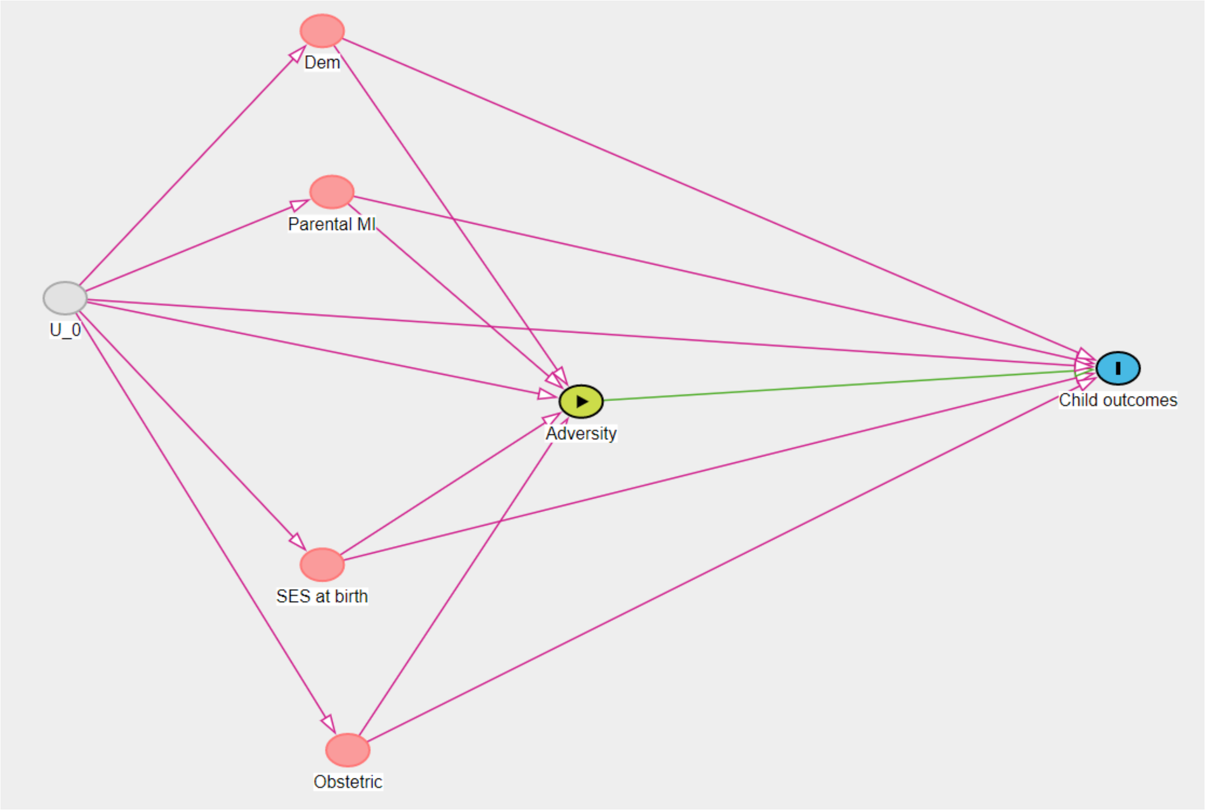

Where:

Dem = measured demographic variables relating to the family: parental country of birth (categorised in to: Swedish; other Scandinavian; African; Asian; European Other; Middle Eastern; Other); parental age at birth; and highest level of parental education (categorised as: secondary, post-secondary and degree).

Parental MI = whether or not a parent had an inpatient hospital event where a mental illness was recorded in the 5 years prior to birth. Parental mental illness has consistently shown to be related to adversities like parental separation or poverty[2] and the risk of poor outcomes[3–6]. In the analysis, a variable was created separately for mothers and fathers and for each categories of:

- common mental disorders: anxiety disorders, including OCD and PTSD (ICD-10 F40–8) or depressive disorders (F32-39);
- serious mental illness: affective psychosis (F25, F30–1) or non-affective psychosis (F20–4, F28–9);
- Other mental disorders: eating disorder (F50–3), personality disorder (F60–3) or ADHD (F90);
- Drug or alcohol misuse disorder (F10-17, F18-19, excluding F1X. 0 ‘acute intoxication’)

When the exposure of interest is the adversity ‘parent with a hospital episode with psychiatric problem’ over childhood then controlling for this variable means that the interpretation is ‘disregarding the effect of mental illness that was known at birth, what would be the effect of a parent having a psychiatric disorder over childhood’.

SES = household disposable income measured during the same year as the birth. Disposable income is calculated by Statistics Sweden as the yearly sum of income and public benefits earned by all family members, adjusted for taxation. This has been equivalized, meaning that the total disposable household income (sum of income for each person in the household) is divided by a weighted sum of people in the house, with individual ‘consumption’ weights derived for each member of the household[7]. There is good evidence that income is related to a range of adversities[8] and also children’s development[9,10]. Similar to parental mental illness, controlling for this in an analysis of poverty over childhood means the aim is to investigate whether poverty over childhood has an effect, over and above longstanding effects of low income. This was considered appropriate as the purpose of the analysis is to identify potential intervention targets over the life-span of a child and not targets for families that are considering having a child.

© 2023 Pierce M et al. *JAMA Network Open.*

Obstetric variables = gestational age and birthweight. Early fetal development has been linked to children's development, including their risk of mental illness[11] and poor school grades[12]. It is less clear that these have a direct effect on adversity, however they are likely intermediaries between unobserved, or poorly measured confounders, such as parental mental illness[13]. Therefore controlling for these variables blocks off the portion of the pathway between U and Y that that is through obstetric variables.

U_0 = Unmeasured time-invariant or baseline variables affecting the likelihood of a family experiencing adversity and children's development. These would include unmeasured characteristics of parents, including intelligence, physical health, and personality. These may be passed on to children through genetic or environmental pathways. They would also include unmeasured environmental exposures, such as housing or pollution levels and unmeasured socio-economic exposures, such as hours worked or material possessions. U also includes factors that are poorly measured in the data, for example parental mental health, which is only measured using hospital data, which is likely to account for 20-50% of psychopathology[14], and does not measure sub-clinical poor mental health. Also parental education is fairly crudely measured, so we would expect residual confounding from this.

© 2023 Pierce M et al. *JAMA Network Open.*

eMethods 2: Further details on underlying assumptions behind the causal model

There are three assumptions necessary for the estimates from the model to be causally interpreted – conditional exchangeability, consistency and positivity.

Assumption 1: conditional exchangeability
Before understanding this assumption, it is helpful to define a potential outcome. A potential outcome is the outcome under different levels of the exposure, in this case childhood adversity, which is defined regardless of whether that child experienced that level of adversity. In notation, for a binary exposure a, this is defined as: $Y^{a=1}$ or $Y^{a=0}$. The conditional exchangeability assumption requires that these values are independent of whether or not a child experienced that adversity, conditional on measured covariates X. Another way of saying this is that any variable that predicts the outcome is equally distributed within adversity exposure levels, conditional on X. This is referred to as the 'no unmeasured confounding' assumption. Formally, this can be written as:

$$Y^a \perp\!\!\!\perp A|X$$

Where Y^a is the potential outcome; and $\perp\!\!\!\perp$ is the independence operator. The validity of this assumption in the current study is highly questionable, as there will be many variables that predict the outcome and not equally distributed within adversity exposure levels, for example genetic factors. These are discussed in the main paper (discussion section) and in eMethods 1 above. Also, given that the method for controlling for confounding uses parametric models, there is an assumption that these have been correctly specified. We note that the observed values were close to those predicted from the g-formula models, which provides some evidence that the models are not substantially miss-specified. Only main effects were included in the regression models used in the g-formula algorithm. Future analysis may consider interactions between confounders, or perhaps model relationships using machine learning methods that require less stringent parametric assumptions.

Assumption 2: consistency
This assumption requires that the potential outcome corresponds to the observed outcome, were a child to receive that level of adversity. Formally, this is written as:

$$Y = Y^a \text{ if } A = a$$

This is translated to mean that there is a clear definition of 'adversity exposure' that could potentially be manipulated to provide different potential outcomes. In our study, we have provided analogous hypothetical interventions corresponding to each of the effect measures (see eMethods 3 below). The plausibility of these are discussed in the main manuscript. We note here that, in our opinion, the hypothetical intervention of 'preventing poverty' is the easiest to map to a real-world intervention, given that is defined based on household income, and could be prevented using household benefit payments. Parental mental illness and parental separation are multi-faceted in their determinants, and therefore it is more difficult to conceive of interventional analogues.

Assumption 3: positivity
This assumption requires that the probability of experiencing an adversity (or not) is non-zero for each stratum defined by the confounding covariates X. In other words:

$$P(A = a|X) > 0$$

We did not examine this assumption directly, but note that the adversities were fairly prevalent in the population, and there were no non-zero cells in any cross tabulation with the baseline covariates.

© 2023 Pierce M et al. *JAMA Network Open.*

eMethods 3: Hypothetical prevention scenarios

The estimates from the g-formula models were analogous to intervention scenarios where childhood adversities were prevented from occurring in the population, at given age-periods. For parental mental illness and parental separation, these involved changing the exposure to zero in all children. For poverty, it involved setting all those who were below the poverty threshold to being at the threshold (i.e. at 50% of the median household income). The table below translates these scenarios to hypothetical intervention strategies for age-groups (early years, early primary, late primary, adolescence) where '0' equates to preventing the adversity exposure and 'n' equates to leaving them to follow their natural course.

	Intervention target		
**Scenario**	**Parental mental illness**	**Poverty**	**Separation**
Prevent parent with mental illness	[0, 0, 0, 0]	[n, n, n, n]	[n, n, n, n]
Prevent household relative poverty	[n, n, n, n]	[0, 0, 0, 0]	[n, n, n, n]
Prevent parental separation	[n, n, n, n]	[n, n, n, n]	[0, 0, 0, 0]
Prevent All	[0, 0, 0, 0]	[0, 0, 0, 0]	[0, 0, 0, 0]
Prevent all at specific ages			
Early years (0 to 3)	[0, n, n, n]	[0, n, n, n]	[0, n, n, n]
Early primary (4 to 7)	[n, 0, n, n]	[n, 0, n, n]	[n, 0, n, n]
Late primary (8 to 11)	[n, n, 0, n]	[n, n, 0, n]	[n, n, 0, n]
Adolescence (12 to 16)	[n, n, n, 0]	[n, n, n, 0]	[n, n, n, 0]

© 2023 Pierce M et al. *JAMA Network Open.*

eTable. Association between baseline covariates and CMD prevalence or school leaving grades

Covariate	CMD prevalence OR [95% CI] Unadjusted	CMD prevalence OR [95% CI] Adjusted*	School leaving grades z-scores Beta [95% CI] Unadjusted	School leaving grades z-scores Beta [95% CI] Adjusted*
Year of birth				
1996	Ref	Ref	Ref	Ref
1997	0.99 [0.95, 1.02]	0.99 [0.96, 1.02]	0.034 [0.024, 0.044]	0.020 [0.011, 0.029]
Age of mother at birth				
<21 years	1.79 [1.66, 1.93]	1.56 [1.42, 1.71]	-0.590 [-0.617, -0.562]	-0.316 [-0.345, -0.286]
19-24	1.26 [1.20, 1.32]	1.23 [1.17, 1.29]	-0.295 [-0.310, -0.280]	-0.179 [-0.194, -0.165]
25-29	Ref	Ref	Ref	Ref
30-39	1.03 [1.00, 1.07]	0.98 [0.94, 1.02]	0.112 [0.101, 0.123]	0.093 [0.082, 0.104]
40+	1.24 [1.12, 1.38]	1.07 [0.96, 1.20]	0.057 [0.022, 0.091]	0.095 [0.062, 0.128]
Age of father at birth				
<21 years	1.99 [1.75, 2.25]	1.36 [1.18, 1.57]	-0.651 [-0.700, -0.602]	-0.224 [-0.272, -0.175]
21-24	1.38 [1.30, 1.46]	1.16 [1.09, 1.24]	-0.292 [-0.312, -0.273]	-0.097 [-0.116, -0.077]
25-29	Ref	Ref	Ref	Ref
30-39	0.98 [0.95, 1.02]	1.05 [1.01, 1.10]	0.150 [0.139, 0.161]	0.059 [0.048, 0.071]
≥40+	1.10 [1.04, 1.17]	1.16 [1.08, 1.24]	0.131 [0.113, 0.149]	0.058 [0.039, 0.077]
Maternal education				
Pre-upper-secondary	Ref	Ref	Ref	Ref
Secondary	0.82 [0.77, 0.86]	0.82 [0.77, 0.87]	0.435 [0.418, 0.453]	0.312 [0.294, 0.329]
Post-upper-secondary	0.75 [0.71, 0.79]	0.80 [0.75, 0.85]	0.985 [0.967, 1.002]	0.653 [0.634, 0.671]
Paternal education				
Pre-upper-secondary	Ref	Ref	Ref	Ref
Secondary	0.87 [0.83, 0.91]	0.90 [0.85, 0.94]	0.295 [0.281, 0.309]	0.194 [0.180, 0.208]
Post-upper-secondary	0.77 [0.73, 0.81]	0.86 [0.81, 0.91]	0.889 [0.873, 0.904]	0.569 [0.553, 0.584]
Country of birth mother				
African	0.39 [0.32, 0.48]	0.43 [0.32, 0.60]	-0.221 [-0.266, -0.176]	0.049 [-0.022, 0.121]
Asian	0.79 [0.70, 0.89]	0.78 [0.68, 0.90]	0.062 [0.028, 0.096]	0.127 [0.089, 0.165]
European other	0.68 [0.62, 0.75]	0.69 [0.61, 0.77]	-0.053 [-0.077, -0.028]	0.012 [-0.017, 0.041]
Middle Eastern	0.60 [0.55, 0.66]	0.54 [0.46, 0.64]	-0.203 [-0.228, -0.178]	0.092 [0.048, 0.136]
Other	1.18 [1.03, 1.36]	0.99 [0.85, 1.15]	-0.147 [-0.194, -0.100]	-0.062 [-0.107, -0.016]
Scandinavian other	1.14 [1.03, 1.26]	1.09 [0.98, 1.20]	-0.105 [-0.137, -0.072]	-0.067 [-0.096, -0.037]
Swedish	Ref	Ref	Ref	Ref
Country of birth father				
African	0.49 [0.40, 0.58]	0.81 [0.61, 1.07]	-0.206 [-0.249, -0.163]	0.008 [-0.060, 0.076]
Asian	0.78 [0.68, 0.90]	0.85 [0.72, 1.01]	0.087 [0.047, 0.127]	0.224 [0.179, 0.268]
European other	0.80 [0.74, 0.87]	0.95 [0.85, 1.05]	-0.081 [-0.105, -0.057]	0.027 [-0.001, 0.055]
Middle Eastern	0.69 [0.64, 0.75]	1.01 [0.87, 1.17]	-0.206 [-0.228, -0.183]	-0.037 [-0.077, 0.004]
Other	1.41 [1.24, 1.59]	1.36 [1.18, 1.56]	-0.159 [-0.203, -0.114]	-0.096 [-0.139, -0.053]
Scandinavian other	1.34 [1.22, 1.48]	1.27 [1.15, 1.40]	-0.195 [-0.228, -0.161]	-0.085 [-0.115, -0.054]
Swedish	Ref	Ref	Ref	Ref
Birth order				
1st	Ref	Ref	Ref	Ref
2nd	0.93 [0.89, 0.96]	0.98 [0.95, 1.02]	-0.128 [-0.139, -0.117]	-0.195 [-0.206, -0.185]
3rd or higher	1.07 [1.02, 1.11]	1.11 [1.05, 1.16]	-0.354 [-0.368, -0.340]	-0.390 [-0.403, -0.376]
Prior parental mental illness*				
None	Ref	Ref	Ref	Ref
Paternal only	1.87 [1.67, 2.10]	1.79 [1.59, 2.00]	-0.415 [-0.458, -0.371]	-0.291 [-0.331, -0.252]
Maternal only	2.30 [2.11, 2.51]	2.13 [1.95, 2.33]	-0.317 [-0.352, -0.282]	-0.191 [-0.223, -0.159]
Both parents	3.76 [2.80, 5.04]	3.18 [2.36, 4.28]	-0.734 [-0.876, -0.592]	-0.423 [-0.551, -0.294]
Household in relative poverty at birth	0.93 [0.88, 0.98]	0.96 [0.90, 1.02]	-0.344 [-0.360, -0.328]	-0.091 [-0.108, -0.074]
Born low birthweight**	1.22 [1.11, 1.33]	1.19 [1.07, 1.32]	-0.143 [-0.173, -0.113]	-0.097 [-0.129, -0.065]
Born preterm***	1.11 [1.03, 1.20]	0.99 [0.91, 1.08]	-0.094 [-0.117, -0.070]	-0.028 [-0.053, -0.002]

Note: estimates obtained from logistic regression model (CMD prevalence) or linear regression model (school leaving grades z-scores); Ref = reference category; *Adjusted for all other variables

© 2023 Pierce M et al. *JAMA Network Open.*

eFigure 1. Prevalence of common mental disorders by adversity exposure

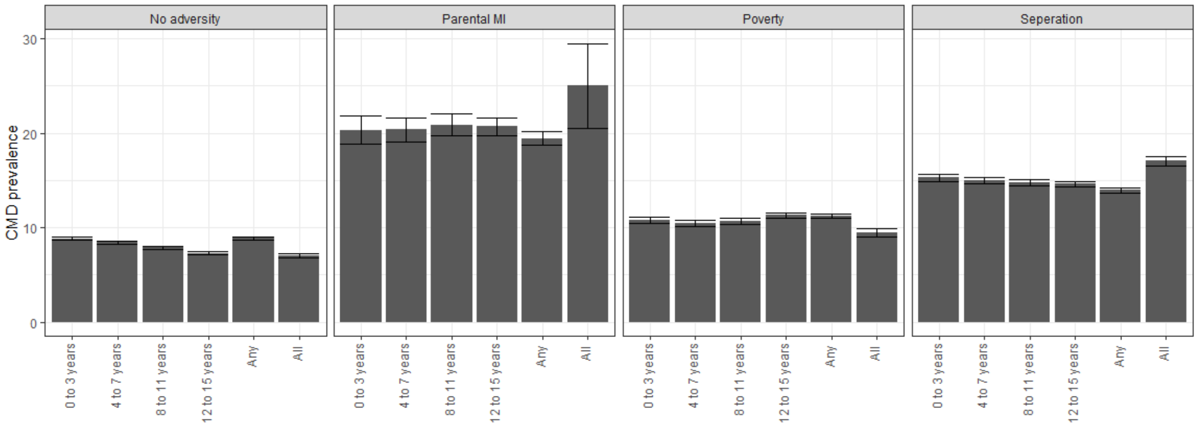

© 2023 Pierce M et al. *JAMA Network Open.*

eFigure 2. Average school grade z-scores by adversity exposure

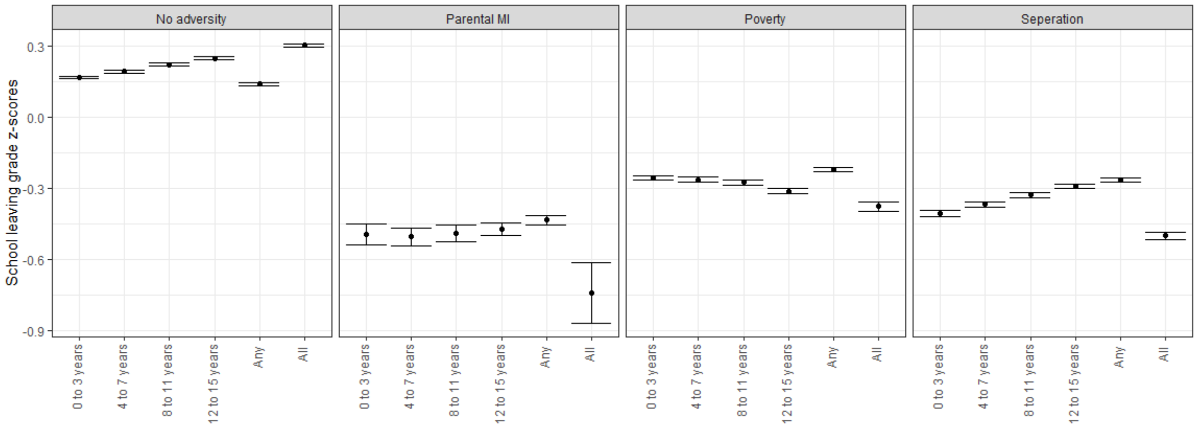

© 2023 Pierce M et al. *JAMA Network Open.*

eReferences

1. Daniel RM. G-Computation Formula. *Wiley StatsRef: Statistics Reference Online*. Published online 2018:1-10. doi:10.1002/9781118445112.stat08030

2. Pierce M, Abel KMKM, Muwonge J, et al. Prevalence of parental mental illness and association with socioeconomic adversity among children in Sweden between 2006 and 2016: a population-based cohort study. *The Lancet Public Health*. 2020;5(11):e583-e591. doi:10.1016/S2468-2667(20)30202-4

3. Shen H, Magnusson C, Rai D, et al. Associations of Parental Depression With Child School Performance at Age 16 Years in Sweden. *JAMA Psychiatry*. 2016;73(3):239. doi:10.1001/jamapsychiatry.2015.2917

4. Lin A, Di Prinzio P, Young D, et al. Academic performance in children of mothers with schizophrenia and other severe mental illness, and risk for subsequent development of psychosis: A population-based study. *Schizophrenia Bulletin*. 2017;43(1):205-213. doi:10.1093/schbul/sbw042

5. Pearson RM, Evans J, Kounali D, et al. Maternal depression during pregnancy and the postnatal period risks and possible mechanisms for offspring depression at age 18 years. *JAMA Psychiatry*. 2013;70(12):1312-1319. doi:10.1001/jamapsychiatry.2013.2163

6. Lewis G, Neary M, Polek E, Flouri E, Lewis G. The association between paternal and adolescent depressive symptoms: evidence from two population-based cohorts. *Lancet Psychiatry*. 2017;4(12):920-926. doi:10.1016/S2215-0366(17)30408-X

7. Ludvigsson JF, Svedberg P, Olén O, Bruze G, Neovius M. The longitudinal integrated database for health insurance and labour market studies (LISA) and its use in medical research. *Eur J Epidemiol*. 2019;(March). doi:10.1007/s10654-019-00511-8

8. Sareen J, Afifi TO, McMillan KA, Asmundson GJG. Relationship Between Household Income and Mental Disorders. *Archives of General Psychiatry*. 2011;68(4):419. doi:10.1001/archgenpsychiatry.2011.15

9. Chevalier A, Harmon C, O' Sullivan V, Walker I. The impact of parental income and education on the schooling of their children. *IZA Journal of Labor Economics*. 2013;2(1):1-22. doi:10.1186/2193-8997-2-8

10. Carod-Artal FJ. Social determinants of mental health. *Global Mental Health: Prevention and Promotion*. Published online 2017:33-46. doi:10.1007/978-3-319-59123-0_4

11. Xia Y, Xiao J, Yu Y, et al. Rates of Neuropsychiatric Disorders and Gestational Age at Birth in a Danish Population. *JAMA Network Open*. Published online 2021:1-13. doi:10.1001/jamanetworkopen.2021.14913

12. Islam MM. The effects of low birth weight on school performance and behavioral outcomes of elementary school children in Oman. *Oman Medical Journal*. 2015;30(4):241-251. doi:10.5001/omj.2015.50

13. Stein A, Pearson RM, Goodman SH, et al. Effects of perinatal mental disorders on the fetus and child. *The Lancet*. 2014;384(9956):1800-1819. doi:10.1016/S0140-6736(14)61277-0

14. Sundquist J, Ohlsson H, Sundquist K, Kendler KS. Common adult psychiatric disorders in Swedish primary care where most mental health patients are treated. *BMC Psychiatry*. 2017;17(1):1-9. doi:10.1186/s12888-017-1381-4

© 2023 Pierce M et al. *JAMA Network Open.*
