# Supplementary material for: Prevention of Childhood Adversities and Children’s Common Mental Disorders and School Grades
Source: JAMA Netw Open. 2023 Oct 5;6(10):e2336408. doi: 10.1001/jamanetworkopen.2023.36408 (PMC10556962; doi:10.1001/jamanetworkopen.2023.36408)
Supplement: Supplement 2. — Data Sharing Statement [file jamanetwopen-e2336408-s002.pdf]

Data Sharing Statement

Pierce. Prevention of Childhood Adversities and Children's Common Mental Disorders and School Grades. *JAMA Netw Open*. Published October 05, 2023.
doi:10.1001/jamanetworkopen.2023.36408

Data
Data available: No
